# Update from the Asia Pacific Malaria Elimination Network (APMEN)

**DOI:** 10.1186/1475-2875-11-S1-O54

**Published:** 2012-10-15

**Authors:** Maxine Whittaker

**Affiliations:** 1 Asia Pacific Malaria Elimination Network (APMEN), Queensland, Australia; 2 Australian Centre for International and Tropical Health (ACITH), University of Queensland, Australia; 3 Pacific Malaria Initiative Support Centre PacMISC, Queensland, Australia

## Background

Community engagement and participation has played a critical role in successful disease control and elimination campaigns in many countries. Despite this, its benefits for malaria control and elimination are yet to be fully realised, and research in this area has been identified by MalERA as a priority. The Pacific Malaria Initiative - a partnership between Vanuatu, Solomon Islands, AusAID, WHO, SPC and in-country partners has been supporting operational and applied research activities to understand effective ways to engage the communities in their programmes. The Asia Pacific Malaria Elimination Network has had a focus of work in community engagement, and draws upon lessons from the country programmes within the region. This paper reports upon some of the challenges faced in elimination, and the tools, approaches and insights gained in the Asia Pacific Region at the implementation level of elimination of malaria.

## Results

The paper will present strategies developed and/or trialled in countries in the Asia Pacific Region to develop sustainable engagement by communities in the targeted locations to maintain and support malaria control activities and be engaged in the identification of malaria cases, and protection of borders. as defined in the national malaria elimination strategy. These include: community participation to reduce transmission and reservoir of infection (including IRS, source reduction, LLINs); community based treatment support for people who are using malaria treatment (*vivax* or *falciparum*) (early recognition of fever, active case detection, directly observed treatment and adherence, community based distribution support, test before treatment behaviour); develop and strengthen community self monitoring of community level surveillance.

## Conclusions

The operational realities of moving towards malaria elimination demonstrate the need to address community participation and engagement., Approaches that have been successful in other elimination and eradication activities may form one set of strategies to trial. Additionally new tools such as GIS can support the sustaining of engagement in these efforts.

